# Evolution and Creationism in America's Classrooms: A National Portrait

**DOI:** 10.1371/journal.pbio.0060124

**Published:** 2008-05-20

**Authors:** Michael B Berkman, Julianna Sandell Pacheco, Eric Plutzer

## Abstract

Despite many legal and legislative decisions, a new study shows that one in eight high school biology instructors teach their students that creationism or intelligent design is a valid alternative to evolutionary biology.

In 2004, the school board in Dover, Pennsylvania, voted to require its 9th grade science teachers to read a statement questioning the validity of evolutionary theory. “Because Darwin's Theory is a theory,” teachers were instructed to say, “it continues to be tested as new evidence is discovered. The Theory is not a fact. Gaps in the Theory exist for which there is no evidence.” Students in Dover High School were also encouraged to explore the concept of intelligent design (ID), described in the statement as “an explanation of the origin of life that differs from Darwin's view.” Multiple copies of the ID text *Of Pandas and People* were made available, and the school board stated that “Students are encouraged to keep an open mind. The school leaves the discussion of the Origins of Life to individual students and their families”[1].

By promoting ID and questioning evolution, Dover's elected school board aligned itself with national public opinion, which consistently shows a majority favors teaching Biblical creationism in addition to evolution [2]. Moreover, a 2005 poll conducted by the Pew Forum on Religion and Public Life reports that 38% of Americans would prefer that creationism was taught instead of evolution [3]. But the Dover public school teachers, citing ethical obligations, were unmoved by public pressure and refused to comply with their board's directive. The high school's science teachers issued a statement arguing:

*“…if I as the classroom teacher read the required statement, my students will inevitably (and understandably) believe that Intelligent Design is a valid scientific theory, perhaps on par with the theory of evolution. That is not true. To refer the students to ‘Of Pandas and People’ as if it is a scientific resource breaches my ethical obligation to provide them with scientific knowledge that is supported by recognized scientific proof or theory”* [1].

To scientists, the teachers' position is noncontroversial. Alternative approaches to evolution like ID are a “hoax” at best and “faith” at worst [4,5]; in neither case do they have any place in a science curriculum. The National Academy of Sciences calls evolution “the central concept of biology” [6], and three respected national organizations have provided model high school curriculum guidelines with evolution as a unifying theme [7–9].

## Teaching Evolution: Law, Policy, and Practice

**Figure 1:**
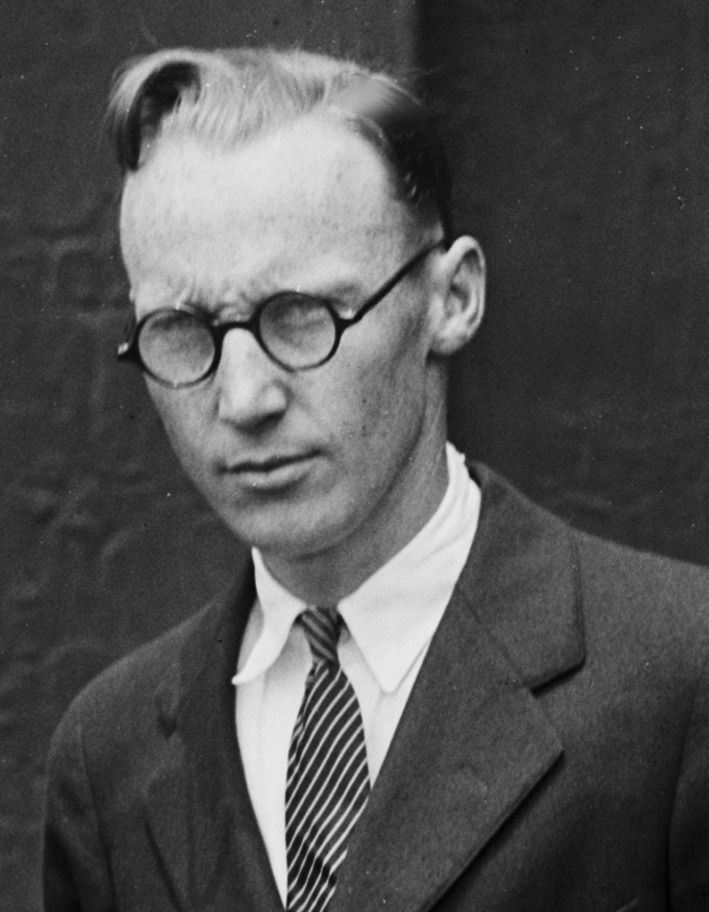
John Scopes On May 7, 1925, John T. Scopes was arrested for teaching evolution at Rhea County High School in Dayton, Tennessee. When the famous “monkey trial” ended, Scopes was convicted of violating a Tennessee law that made it a crime to “teach any theory that denies the story of the Divine Creation of man as taught in the Bible, and to teach instead that man is descended from a lower order of animals.” Since that time, teachers have been on the front lines of the battles between evolutionary biology and alternatives such as intelligent design and creationism.

Unlike John Scopes (see Figure 1), the Tennessee biology teacher convicted of teaching evolution (a conviction upheld in the 1925 case of *Tennessee v. John Scopes*), the plaintiffs and teachers in Dover prevailed in the courts when the Dover classroom disclaimer was declared unconstitutional. Consistent with earlier cases in other states, the court in *Kitzmiller v. Dover* found that ID—like other more explicitly religious alternatives to evolution—must be excluded from public school classrooms as a violation of the Constitution's Establishment Clause [10,11]. Judge John E. Jones III's ruling could not have been stronger: the Dover school board's actions were of “breath-taking inanity” and an “utter waste of monetary and personal resources [1].”

Victories in cases like *Kitzmiller* are important to the scientific community, which devotes time and resources to exclude the teaching of nonscientific alternatives to evolutionary theory. These victories have paid dividends in policies at the state and local level. Although the United States has no national curriculum guidelines or requirements in any area of science, state governments do. These standards provide local school boards within each state with a common guide to classroom instruction in science and other subjects. While these standards vary widely in quality and detail from state to state, all recognize, at least to some degree, the importance of evolutionary theory. At this time, not a single state uses its content standards to explicitly promote ID or creationism [12–14]. School boards are monitored by organizations like the National Center for Science Education, by state academies of science, and by local scientific and professional organizations. As a result, few state school boards can formally consider measures like the one adopted in Dover without scrutiny and challenge from organizations representing the scientific profession.

These legal rulings and legislative victories are clearly necessary for evolution to maintain its proper place in the biology curriculum, but they are not sufficient. Implementation of state standards, adherence to court decisions, and the full integration of textbook material rests in the hands of the thousands of classroom teachers throughout the country. And about this, we are less sanguine. Notwithstanding the professionalism and bravery of the teachers in Dover, the status of evolution in the biology and life sciences curriculum remains highly problematic and threatened. Evolution—more precisely opposition to it—is profoundly important to fundamentalist Christianity, where it has played a critical role in its early formation as doctrine and as a social movement [15,16]. Within American politics generally, religious-based conflict is increasingly salient [17]; even President Bush has expressed support for teaching “both sides” of the evolution controversy. But opposition to evolution can be especially intense at the local level, where teachers live and work. This may occur through the election of “stealth” school board candidates [18], or when teachers face organized and unorganized opposition and questioning of their curriculum from religiously motivated members of the community [19,20].

Community pressures place significant stress on teachers as they try to teach evolution, stresses that can lead them to de-emphasize, downplay, or ignore the topic [20]. This is particularly true of the many teachers who lack a full understanding of evolution, or at least confidence in their knowledge of it. Such a lack of confidence can lead teachers to avoid confrontations with students, parents, and the wider community. They may, for example, not treat evolution as the class's organizing principle, or may avoid effective hands-on activity to teach it, or not ask students to apply natural selection to real life situations [19]. There are many reasons to believe that scientists are winning in the courts, but losing in the classroom. This is partially due to the occasional explicit teaching of creationism and ID, but most especially because of inconsistent emphasis and minimal rigor in the teaching of evolution.

Evolution—more precisely opposition to it—is profoundly important to fundamentalist Christianity, where it has played a critical role in its early formation as doctrine and as a social movement.

Studies of science teachers seem to confirm these fears by suggesting “that instruction in evolutionary biology at the high school level has been absent, cursory or fraught with misinformation” [21]. But we are wary of this conclusion. Most of the previous studies are now dated; the recent ones each examine a single state, and many states (most notably California, New York, and all of New England) have never been studied (see [19,21,22] for comprehensive reviews of these single-state studies). Collectively, the studies employ incomparable measures, and some of them sacrificed scientific sample survey methods in favor of higher cooperation rates (such as surveys of teachers attending conventions and professional meetings [23]). As a result, we lack a systematic and coherent account of how instruction varies from teacher to teacher across the nation as a whole. To remedy this, we provide a statistical portrait of evolution and creationism in America's classrooms, from which we draw conclusions about the unevenness of how evolutionary biology is taught and some of the causes of that variation.

## The National Survey of High School Biology Teachers

We advance this long tradition of surveying teachers with reports from the first nationally representative survey of teachers concerning the teaching of evolution. The survey permits a statistically valid and current portrait of US science teachers that complements US and international surveys of the general public on evolution and scientific literacy [2,24] and on evolution in the classroom [3,25]. Between March 5 and May 1, 2007, 939 teachers participated in the study, either by mail or by completing an identical questionnaire online. Our overall response rate of 48% yielded a sample that may be generalized to the population of all public school teachers who taught a high school–level biology course in the 2006–2007 academic year, with all percentage estimates reported in this essay's tables and figures having a margin of error of no more than 3.2% at the 95% confidence level. Detailed discussion of the methods of the survey and assessments of non-response can be found in Text S1. Our results confirm wide variance in classroom instruction and indicate a clear need to focus not only on state and federal policy decisions, but on the everyday instruction in American classrooms.

*Evolution in the classroom*: How much time should be spent on evolution in the typical high school biology class? There is no clear answer to this question. Neither the strongest nor the weakest state standards specify a precise amount of time that should be spent on any particular topic. As we noted above, there are three widely circulated documents that serve as guidelines at the national level [6–8], but these, too, refrain from offering directions on the amount of time that should be spent on evolution relative to other topics. In general, these national reports and state standards offer ideas for the content of high school science, biology, and life science classes, but not the curriculum; in other words, they enumerate and elaborate on outcomes—what students should learn—but not on any particular ordering or allocation of time for each subject.

It is clear, however, that all three of these reports expect and recommend a substantial investment in evolutionary biology and evolution-related topics. All expect science teachers to “provide evidence that evolution has attained its status as a unifying theme in science” [12]. The National Research Council's 1996 *National Science Education Standards* (NSES), often used as a benchmark to evaluate the content of state science standards and textbooks, identifies evolution as one of the five “unifying concepts and processes” that provide the “big picture of scientific ideas.” The NSES further identifies 11 benchmarks (for example, natural selection, biological adaptation) for states and textbook editors to use in determining the content for high school biology materials.

Community pressures place significant stress on teachers as they try to teach evolution, stresses that can lead them to de-emphasize, downplay, or ignore the topic.

We followed most previous studies in asking teachers to think about how they allocate time over the course of the school year. We went a step further in also asking whether evolution serves as a unifying theme for the content of the course. Over the entire year of high school biology we found substantial variation among America's high school teachers (see Table 1). Not surprisingly, we found that those who take most seriously the advice of NSES to make evolution a unifying theme spent the most time on evolution. Overall, teachers devoted an average of 13.7 hours to general evolutionary processes (including human evolution), with 59% allocating between three and 15 hours of class time (see Table S1). Only 2% excluded evolution entirely. But significantly fewer teachers covered human evolution, which is not included as an NSES benchmark. Of teachers surveyed, 17% did not cover human evolution at all in their biology class, while a majority of teachers (60%) spent between one and five hours of class time on it.

**Table 1:**
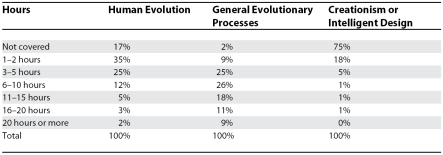
Hours Devoted to Human Evolution, General Evolution, and Creationism or Intelligent Design in High School Biology Classes, 2007 (*n* = 939)

Those teachers who stressed evolution by making it the unifying theme of their course spent more time on it. Overall, only 23% strongly agreed that evolution served as the unifying theme for their biology or life sciences courses (Table S2); these teachers devoted 18.5 hours to evolution, 50% more class time than other teachers. When we asked whether an excellent biology course could exist without mentioning Darwin or evolutionary theory at all, 13% of teachers agreed or strongly agreed that such a course could exist.

*Creationism in the classroom*: We also asked teachers whether they spent classroom time on creationism or intelligent design. We found that 25% of teachers indicated that they devoted at least one or two classroom hours to creationism or intelligent design (see Table 1). However, these numbers can be misleading because while some teachers may cover creationism to expose students to an alternative to evolutionary theory, others may bring up creationism in order to criticize it or in response to student inquiries. Questions that simply ask about time devoted to creationism, therefore, will overstate support for creationism or intelligent design by counting both those who teach creationism as a serious subject and those holding it up for criticism or ridicule. We asked a series of supplemental questions that provided some additional insight into the character of creationism in the classroom. Of the 25% of teachers who devoted time to creationism or intelligent design, nearly half agreed or strongly agreed that they teach creationism as a “valid scientific alternative to Darwinian explanations for the origin of species.” Nearly the same number agreed or strongly agreed that when they teach creationism or intelligent design they emphasize that “many reputable scientists view these as valid alternatives to Darwinian Theory” (see Table S3).

On the other hand, many teachers devoted time to creationism either to emphasize that religious theories have no place in the science classroom or to challenge the legitimacy of these alternatives. Of those who spent time on the subject, 32% agreed or strongly agreed that when they teach creationism they emphasize that almost all scientists reject it as a valid account of the origin of species, and 40% agreed or strongly agreed that when they teach creationism they acknowledge it as a valid religious perspective, but one that is inappropriate for a science class.

*Explaining differences in teachers' emphasis*: Why do some teachers spend so much more time on evolution than others? Our data weigh heavily against one possible explanation: differences in state standards. We find that nearly 90% of cross-teacher variation is within states (Eta-square from a one-way analysis of variance by state is 0.11) as opposed to between states. As an upper limit, then, state standards cannot account for more than 11% of the variance [21].

However, our data lend support to two potential explanations: teachers' personal beliefs about evolution and the number of college-level science classes.

**Figure 2:**
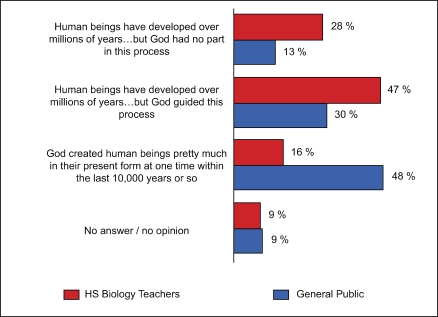
High School Biology Teachers' Personal Beliefs Concerning Human Origins, Compared with a Representative Sample of the General Public, Spring 2007 Notably, we find that teachers' personal beliefs are linked to classroom instruction. The teachers who chose the “young earth” creationist position devoted 35% fewer class hours to evolution (9.6 hours) than all other teachers (14.7 hours).

Our teachers were each asked a question about their own personal beliefs about human origins. This question is identical to a question that major polling organizations have asked members of the general public since 1981 [2]. Figure 2 compares the results for our sample of teachers surveyed during March and April of 2007 with the results of a public opinion poll conducted for *Newsweek* on March 28–29 of 2007 (see Table S4). Among the biology teachers, 16% believed that human beings were created by God in their present form at one time within the last 10,000 years (and an additional 9% declined to answer). Although this is a far smaller proportion than found among the general public (48%), our data demonstrate substantial sympathy for the “young earth” creationist position among nearly one in six members of the science teaching profession. The teachers who chose the “young earth” creationist position devoted 35% fewer class hours to evolution than all other teachers (Table S5).

*Teacher qualifications*: The No Child Left Behind Act requires that all teachers of core subjects be “highly qualified.” Definitions of “highly qualified” vary by state, but most include demonstrated competence in the teacher's teaching assignment. Our data suggest that high school teachers who completed the largest number of college-level credits in biology and life science classes and whose coursework included at least one class in evolutionary biology devote substantially more class time to evolution than teachers with fewer credit hours (Table S6). The best prepared teachers devote 60% more time to evolution than the least prepared.

## Evolution in the Classroom? It's about the Teachers

Our survey of biology teachers is the first nationally representative, scientific sample survey to examine evolution and creationism in the classroom. Three different survey questions all suggest that between 12% and 16% of the nation's biology teachers are creationist in orientation. Roughly one sixth of all teachers professed a “young earth” personal belief, and about one in eight reported that they teach creationism or intelligent design in a positive light. The number of hours devoted to these alternative theories is typically low—but this nevertheless must surely convey to students that these theories should be accorded respect as scientific perspectives.

The majority of teachers, however, see evolution as central and essential to high school biology courses. Yet the amount of time devoted to evolutionary biology varies substantially from teacher to teacher, and a majority either avoid human evolution altogether or devote only one or two class periods to the topic. We showed that some of these differences were due to personal beliefs about human origins. However, an equally important factor is the science education the teacher received while in college. Additional variance is likely to be rooted in pressures—subtle or otherwise—emerging from parents and community leaders in each school's community, in combination with teachers' confidence in their ability to deal with such pressures [20] given their knowledge of evolution, as well as their personal beliefs.

These findings strongly suggest that victory in the courts is not enough for the scientific community to ensure that evolution is included in high school science courses. Nor is success in persuading states to adopt rigorous content standards consistent with recommendations of the National Academy of Sciences and other scientific organizations. Scientists concerned about the quality of evolution instruction might have a bigger impact in the classroom by focusing on the certification standards for high school biology teachers. Our study suggests that requiring all teachers to complete a course in evolutionary biology would have a substantial impact on the emphasis on evolution and its centrality in high school biology courses. In the long run, the impact of such a change could have a more far reaching effect than the victories in courts and in state governments.

## Supporting Information

Table S1 Mean Hours Devoted to Human Evolution, General Evolution, and Creationism or Intelligent Design in High School Biology Classes, 2007 Teachers selected categories in order to indicate the number of hours devoted to evolution and creationism (see Table 1). Using category midpoints, and assuming a mean of 25 hours for the last category (22 hours and more) we calculated the mean number of hours devoted to each of these three topics. The means are referred to in the text and are reported here.
(18 KB XLS).

Table S2 Teacher Reports on the Centrality of Evolution in High School Biology Classes (16 KB XLS).

Table S3 Teacher Orientations to Discussing Creationism or Intelligent Design in High School Biology Classes The data here are restricted to 224 teachers who reported spending one or more hours of class time on creationism or intelligent design.
(19 KB XLS).

Table S4 Comparison of Personal Beliefs about Human Origins Held by a Random Sample of US Adults and Our Sample of US High School Biology Teachers The source for the general public is a *Newsweek* poll conducted by Princeton Survey Research Associates International, March 28–29, 2007. The data are archived at the Roper Center for Public Opinion Research, data set USPSRA2007-NW05.
(18 KB XLS).

Table S5 Mean Number of Classroom Hours Devoted to Human Evolution and General Evolution (Combined), By Expressed Personal Beliefs about Human Origins (17 KB XLS).

Table S6 Mean Number of Classroom Hours Devoted to Human Evolution and General Evolution (Combined), By Number of College-Level Biology Credits and Whether the Teacher Completed a Class Devoted to Evolution (17 KB XLS).

Text S1 Materials and Methods (27 KB DOC).

## Abbreviations

ID: intelligent design
NSES: National Science Education Standards
