# Supplementary material for: Evolution and Creationism in America's Classrooms: A National Portrait
Source: PLoS Biol. 2008 May 20;6(5):e124. doi: 10.1371/journal.pbio.0060124 (PMC2386841; doi:10.1371/journal.pbio.0060124)
Supplement: Supplementary file 1 [file pbio.0060124.sd001.pdf]

Materials and Methods

Sampling methods

The data for this paper come from a survey of high school biology teachers conducted between March 5 and May 1, 2007. More precisely, the data are from two simultaneous studies using identical questionnaires and overlapping sampling frames. One study was a mail-only study with teachers selected randomly from a database maintained by Quality Education Data. The database contains names and school mailing addresses for more than 80% of public school teachers in the United States. To be eligible for selection, teachers needed to be in a public school that included grades 9 and 10. Each teacher had from one to six job descriptors in the database; to be eligible for selection at least one descriptor has to be "biology," "life sciences," or "AP biology." This meant that a small number of teachers in our sample identified their primary job function as outside of biology; about 2% identified their primary field as in another science or in science support (e.g., computer lab coordinator) and 12% identified themselves as chair of their high school's science department. Those teachers recruited to teach biology from non-scientific fields were, therefore, eligible for inclusion but comprise a trivial proportion of the sample.

Following the *Tailored Design Method* for mail surveys [1], five hundred teachers received a pre-notification letter, a survey packet (with a two dollar bill and postage paid return envelope), a reminder postcard, and a replacement packet. We received 200 completed questionnaires for a return rate of 40%.

The second study is based on 1,500 names drawn from a *subset* of the original database based on the availability of a working email address. This allowed us to not

only include all features from the first study but also two additional email follow-up reminders. These emails included a link to a web version of the survey, making this survey “multimodal” [1,2]. A total of 739 respondents completed the multi-modal study for a return rate of 49%. Respondents from both surveys are combined in all analyses reported in this paper. After excluding 46 “out of scope” respondents (e.g., bad address, no longer teaching, not a biology teacher), the response rate for the combined data set is 48% (939/1954).

Response rate in context

Response rates for most kinds of surveys in nearly every academic discipline have declined in the last two decades [3–5]. The same is true for surveys of teachers. Gallup’s *Phi Delta Kappa Survey of Teachers* is not a scholarly research project but is nevertheless illustrative of mail surveys of teachers conducted by the best survey research organizations. The 1986 survey mailed a questionnaire to 2000 teachers, sent a single reminder postcard, and achieved a response rate of 42%. The *same methodology* produced response rates of 26% in 1996, and 18% in 1999. Carefully executed academic surveys with extensive follow-ups (like ours) have yielded response rates somewhat higher. Hess, Maranto and Millman’s 1995 mail survey of California teachers, for example, yielded a response rate of 42% [6].

However, the survey research field has moved away from response rates as the primary indicator of validity and unbiasedness [7]. Indeed, a number of empirical assessments have demonstrated that parameter bias does not necessarily increase as response rates decline [8,9]. Rather, it is preferred to make direct estimates of response bias and of how well the sample reproduces known population parameters for key social

and demographic variables. As we note below, the 939 returned questionnaires are representative of the population of inference and results can be generalized to that population with confidence.

Representativeness of the Sample

The combined data set contains teachers from 49 states (no teachers from Wyoming) and the District of Columbia. We examined the combined sample to determine if it was representative of the population of inference. Although there is no census of teachers, the Common Core of Data (CCD) compiled by the National Center for Educational Statistics is a census of schools and we used this to see if the schools that our sample members taught in were representative of high schools nationally. Our teachers taught at schools with an average enrollment of 1,311 students, compared to the national average of 1,332. Similarly, our sample's schools had an average of 26% qualifying for free lunch as compared to 28% nationally. Our sample under-represents teachers at schools with more than 10% Black (27% compared with 42% nationally) and more than 10% Hispanic (22% versus 39%). In addition, we slightly over-represent Midwestern and small town schools.

Post-stratification weighting

Following standard practice for adjusting surveys for differential non-response across groups, we calculated post-stratification weights to adjust the survey for these [7,10,11]. However, the weighted and unweighted results never differ by more than 2.5% and the same substantive conclusions emerge with either weighted or unweighted analyses. Weighted data are employed for all analyses in this paper.

The Questionnaire

Teachers completed a six page survey containing questions about the content of their most recently taught biology course, more specific questions about the teaching of evolution in particular, and a variety of background questions. The questionnaire was designed in the context of previous studies and we adapted question wording from other studies whenever possible and appropriate. The final questionnaire was six pages long and took about 15 minutes to complete.

References

1. Dillman DA (2000) Mail and internet surveys: The tailored design method. New York: Wiley. 544 p.

2. Mokdad AH, Stroup DF, Giles WH; Behavioral Risk Factor Surveillance Team (2003) Public health surveillance for behavioral risk factors in a changing environment. Recommendations from the Behavioral Risk Factor Surveillance Team. MMWR Recomm Rep 52: 1-12.

3. Biener L, Garrett C, Gilpin E, Roman A, Currivan D (2004) Consequences of declining survey response rates for smoking prevalence estimates. Am J Prev Med 27: 254-257.

4. Burden BC (2000) Voter turnout and the National Election Studies. Political Analysis 18: 389-398.

5. Singer E (2006) Introduction: Nonresponse bias in household surveys. Public Opin Q 70: 637-645.

6. Hess FM, Maranto R, Milliman S (2000) Resistance in the trenches: What shapes teachers' attitudes toward school choice? Educational Policy 14: 195-213.

7. Groves RM, Fowler F, Wiley J , Couper MP, Lepkowski JM, et al. (2004) Survey methodology. New York: Wiley. 448 p.

8. Keeter S, Miller C, Kohut A, Groves R, Presser S (2000) Consequences of reducing nonresponse in a National telephone survey. Public Opin Q 64: 125–48.

9. Merkle D, Edelman M (2002) Nonresponse in exit polls: A comprehensive analysis. In: Groves R, Dillman D, Eltinge J, Little R, editors. Survey nonresponse. New York: Wiley. pp. 243–58.

10. Kish L (1965) Survey sampling. New York: Wiley. 664 p.

11. Lohr S (1999) Sampling: Design and analysis. New York: Duxbury. 512 p.
